# Membrane Core-Specific Antimicrobial Action of Cathelicidin LL-37 Peptide Switches Between Pore and Nanofibre Formation

**DOI:** 10.1038/srep38184

**Published:** 2016-11-30

**Authors:** Mahdi Shahmiri, Marta Enciso, Christopher G. Adda, Brian J. Smith, Matthew A. Perugini, Adam Mechler

**Affiliations:** 1 La Trobe Institute for Molecular Science, La Trobe University, Australia

## Abstract

Membrane-disrupting antimicrobial peptides provide broad-spectrum defence against localized bacterial invasion in a range of hosts including humans. The most generally held consensus is that targeting to pathogens is based on interactions with the head groups of membrane lipids. Here we show that the action of LL-37, a human antimicrobial peptide switches the mode of action based on the structure of the alkyl chains, and not the head groups of the membrane forming lipids. We demonstrate that LL-37 exhibits two distinct interaction pathways: pore formation in bilayers of unsaturated phospholipids and membrane modulation with saturated phospholipids. Uniquely, the membrane modulation yields helical-rich fibrous peptide-lipid superstructures. Our results point at alternative design strategies for peptide antimicrobials.

Multidrug-resistant bacteria pose a looming threat to public health worldwide^1^, and yet there is a steady decrease in the number of new antibiotic drugs undergoing clinical trials^2^. Antimicrobial peptides (AMPs) that provide innate immunity would offer an alternative to traditional antibiotics if their mechanism of action is understood^3^. As such, small AMPs of amphibian and insect origin have been studied extensively with the aim of developing these compounds into antimicrobial drugs^3^,^4^. Likewise, mammals can also secrete antimicrobial peptides, which offer the potential of ‘humanized’ scaffolds for microbial intervention^5^.

In humans, natural killer cells, human neutrophils, and mast cells express and store Cathelicidin precursor protein hCAP18^6^. When cleaved by proteinase 3, the C-terminal 37 residue peptide of hCAP18 becomes a potent antimicrobial agent known as LL-37^7^. LL-37 is active against bacteria, fungi and viruses from ~1 μM effective concentration^8^,^9^. LL-37 is a cationic amphipathic peptide^10^; it contains two helical regions separated by a loop, and an unstructured C-terminal tail^11^,^12^. The biological function of LL-37 is debated; while several studies focus on its direct antimicrobial action, it is frequently described as a skin protector^5^ and it is involved in wound healing^6^.

In its direct antibacterial role, it is believed that LL-37 acts *via* disrupting the bacterial membrane^13^. Generally membrane disrupting AMPs are assumed to act *via* one of three mechanisms of action: (i) formation of a pore with a barrel-stave conformation, where a tight bundle of amphiphilic peptides forms a hydrophilic pore across the membrane, (ii) toroidal pore formation, where a loose bundle of peptides modulates the membrane into a lipid headgroup-lined pore, and (iii) the carpet mode, where peptides remain on the surface of the membrane until a threshold is reached to facilitate a breakdown in membrane integrity^14^,^15^,^16^. However, the mechanism of action of LL-37 does not fit into any of these categories; it remains parallel to the surface throughout its action and does not insert into the membrane^10^, and its orientation is unaffected by peptide concentration, membrane charge, presence of ions, or temperature^17^. Furthermore, LL-37 is not as selective as other α-helical, amphipathic AMPs; it does not exhibit a clear preference for charged membranes^18^ and while its minimum inhibitory concentration (MIC) ranges from 1 to 10 μM for a variety of Gram positive and Gram negative bacteria, it exhibits eukaryotic cytotoxicity at 13–25 μM concentrations^19^,^20^. Hence, it was proposed that LL-37 is a nonspecific, albeit highly effective, cell killer that acts *via* the carpet mechanism^21^,^22^. However, it was shown that LL-37 disrupts the lipid bilayer without breaking the membrane into small fragments, and fluorescence measurements also suggested a pore forming mechanism^23^,^24^. The activity against mammalian cell membranes is also ambiguous: it was proposed that LL-37 could act, at least in part, by decreasing the fluidity and hence lowering the permeability of epithelial cell membranes, making it harder for certain bacteria to attack^25^. Hence, there are many uncertainties around the mechanism of LL-37 action and attention has shifted to developing more active variants of LL-37 using systematic mutation^26^,^27^ while the study of the actual mechanism of action has been largely neglected. In this work, the mechanism of action of LL-37 is probed *in vitro* using a comprehensive biophysical approach centred on the combination of a biomimetic membrane platform^28^ with the quartz crystal microbalance fingerprinting method^29^.

## Molecular Dynamic Simulations of LL-37 Aggregation

In previous studies, LL-37 action was linked to oligomerization^30^; it had been reported that LL-37 can oligomerize on the surface of PC vesicles, while it is mostly monomeric on negatively charged membranes^22^,^30^. It was also suggested that LL-37 can form amyloid-like fibrils^31^. Hence, aggregation is likely a key feature of LL-37 action, and equally likely a prerequisite for understanding the mechanism of action, particularly whether the peptide binds the membrane in a monomeric or oligomeric form. Accordingly we initially performed computer simulations to assess the propensity of the peptide toward aggregation, and to identify the common aggregation geometries.

**Figure 1:**
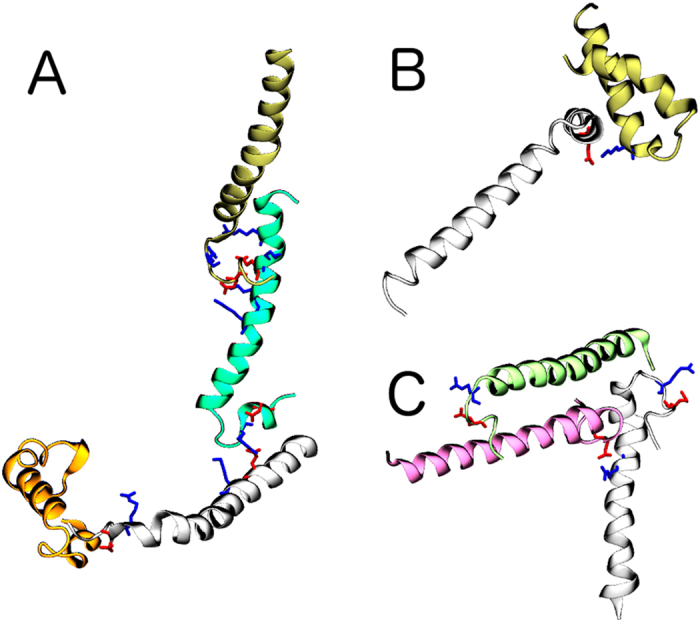
Examples of clustering from MD simulations. The aggregation occurs via salt bridges. (**A**), fibrillar aggregate formed via mostly near-terminal residues; (**B**), attachment mid-sequence; (**C**), trimer aggregate with attachment at terminal and mid-sequence sites.

Atomistic simulations of several LL-37 peptides in a neutral solvated box were conducted. Firstly, it was confirmed that the peptides showed the characteristic helical fold with an unstructured C-terminus^11^,^12^. It was also observed that most of the peptides spontaneously formed intermolecular interactions after ~15 ns, which remained stable until the end of the simulation (50 ns). Most of these interactions were salt bridges involving amino acids in the N- and C-termini; in particular we found that residue D36, in the C terminus, was present in all the detected salt bridges. The structure of the aggregates was mostly unspecific helical clustering of two or three peptides. However, regular fibrous assemblies were also observed, as shown in Fig. 1, indicating the strong potential of LL-37 to form fibrillar aggregates.

It has been suggested previously that α-helical LL-37 peptides oligomerize in solution *via* either the Hofmeister effect^22^,^32^ or the stacking of the aromatic side-chains of residues F5 and F6^33^. Our results suggest that the aggregation is not related to either of these effects, rather to the formation of salt bridges.

## Stages of Peptide-Membrane Interaction: Viscoelastic Fingerprinting

**Figure 2:**
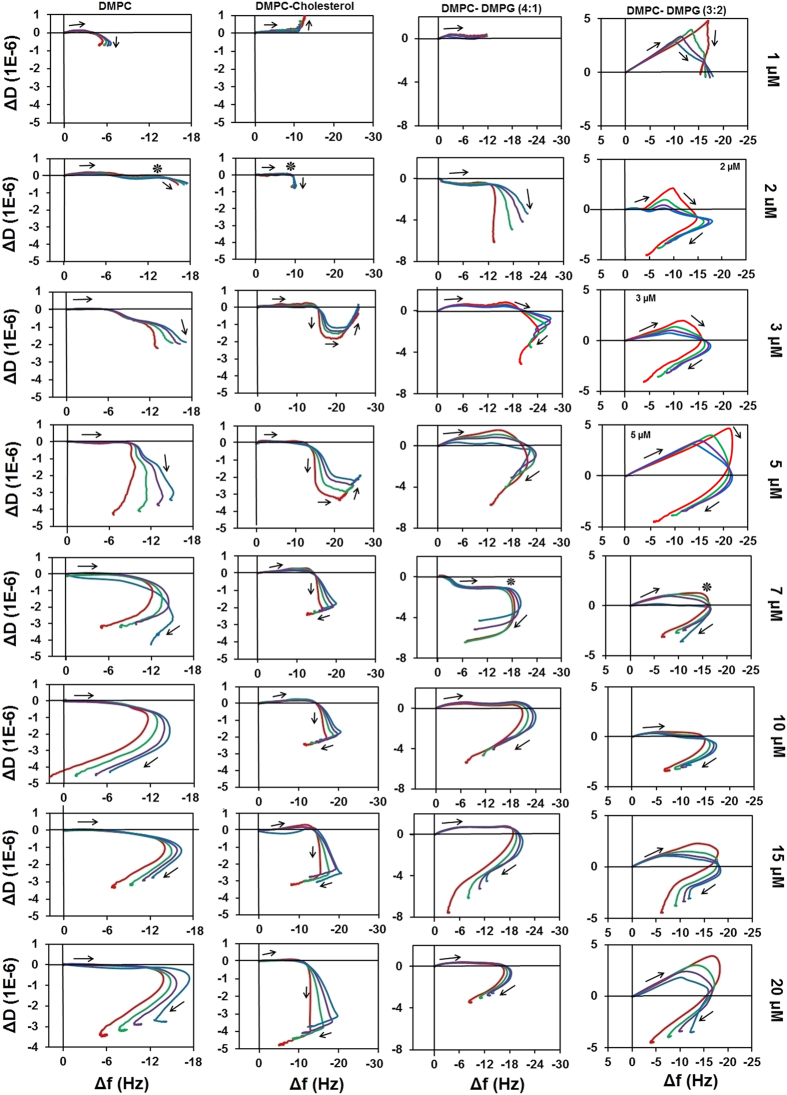
f-D plots of LL-37 interaction with different saturated lipid membranes at peptide concentrations as indicated. f-D fingerprints are shown for the third (red), fifth (green), seventh (purple), and ninth (blue) eigenmodes of the sensor chip. Arrows indicate stages of the interaction; asterisks indicate point of maximal peptide mass bound to charged membranes.

1,2-dimyristoyl-sn-glycero-3-phosphocholine (DMPC) with or without cholesterol is a widely used model for neutral membranes in the literature of AMP membrane interactions, while DMPC mixtures with 1,2-dimyristoyl-sn-glycero-3-phosphoglycerol (DMPG) are frequently used to model charged membranes^34^; hence these lipid mixtures were employed in this study. The interaction of LL-37 with supported phospholipid bilayers of DMPC, DMPC/DMPG (4:1), DMPC/cholesterol (9:1) and DMPC/DMPG (3:2) compositions was investigated using QCM following an established method^29^,^35^. Supported single bilayer membranes had been deposited on MPA functionalized surfaces of the QCM chips. Peptide solutions were introduced to the measurement chambers at systematically varied concentrations ranging from 1 μM to 15 μM; the resulting Δf and ΔD sensograms were checked for irregularities and the results were analysed using the dissipation change versus frequency change (f-D) curves^29^,^35^ as shown in Fig. 2. While the frequency and dissipation sensograms do not reveal mechanistic details directly (Ext. Data Fig. 1, in the f-D fingerprints distinct stages of the peptide-lipid interaction mechanism are apparent as changes in the direction of the curve as indicated with arrows in Fig. 2. Importantly, time is eliminated in this representation and thus these stages reflect processes with different viscoelastic characteristics.

The amount of peptide binding is often reported as its “affinity” to a certain membrane type^36^. LL-37 can bind both neutral and charged membranes, although with a different affinity, as revealed by the initial negative frequency change observed in all f-D curves (Fig. 2). For the zwitterionic membranes, the maximum binding amount was approximately −12 Hz (indicated with asterisk in Fig. 2) irrespective of the peptide concentration or the presence of 10% cholesterol. Maximal binding is reached at 2 μM. With the charged membranes, the binding increased to −18–−22 Hz, and this value is reached at 7 μM; however, the maximal binding did not depend on the percentage of the charged lipid content of the membrane.

F-D plots of the membrane binding of monomeric peptides exhibit a characteristic [−f, +D] slope where the increased energy dissipation is caused by the “rolling” or “wobbling” of the peptides on the membrane surface^35^,^37^. In contrast to other surface acting peptides, LL-37 shows this characteristic slope only in the 3:2 DMPC/DMPG mixture; in the other lipids the process yields negligible dissipation change. It is likely that the high charge and/or the OH moieties of the phosphatidyl glycerol headgroups of the DMPC/DMPG 3:2 membrane are able to compete for the salt bridges that stabilize the aggregates, yielding a mostly monomeric peptide population on the surface; this is consistent with literature reports^22^,^30^. The absence of a dissipative process for all other lipids suggests that the peptide binds the surface as a preassembled aggregate, consistent with the MD simulation results.

The binding stage is followed by further viscoelastic changes in all cases. For neat DMPC, at 3 μM a trendline appears in [−f, −D] and from 7 μM in [+f, −D] direction (stage two) that does not change any further with increased peptide concentration. However, 10% (mol/mol) cholesterol content lead to remarkably different characteristics. At low peptide concentration the fingerprints reveal binding and a complex structural rearrangement in multiple stages that does not lead to appreciable membrane disruption; the trend, however, changes into a three-stage mechanism at 7 μM. It was suggested in the literature that the liquid ordered phase is essential in weakening membrane association of LL-37 and preserving the membrane of host cells from the permeabilizing attack of the peptide^31^, and that cholesterol is known to impose order on the membrane core in the liquid phase^38^. Therefore, the observed differences in the mechanistic fingerprint are consistent with the protective function of LL-37.

In DMPC/DMPG (4:1) the a second stage starts at 2 μM with a [−f, −D] trendline, and increasing the peptide concentration to 3 μM yields a third stage trending to [+f, −D]; above 7 μM peptide concentration the fingerprint shows the same “hook” pattern as in neat DMPC. In DMPC:DMPG (3:2) the first stage shows a more conventional [−f, +D] trend as discussed above, however the overall trend is the same as in DMPC:DMPG (4:1) and the maximum binding is not affected by the increased membrane charge. Thus, all characteristic differences between neat DMPC, DMPC:DMPG (4:1) and DMPC:DMPG (3:2) are observed at peptide concentrations below 7 μM, while above this concentration a very similar hook pattern is observed.

The ability of LL-37 to bind to neutral (mammalian) membranes is controversial in light of its primary antimicrobial function. From earlier studies it is clear that LL-37 interacts favourably with bacterial membranes at lower concentrations^30^. It is believed that electrostatic interactions target cationic AMPs to bacterial membranes^39^. LL-37 has a formal charge of +6 under physiological conditions, thus it is expected to exhibit a stronger binding affinity to anionic lipids^40^ supposedly in proportion to the charge of the membrane. A surprising result from the fingerprinting is that the amount of anionic lipid in the membrane has no influence on the binding affinity.

**Figure 3:**
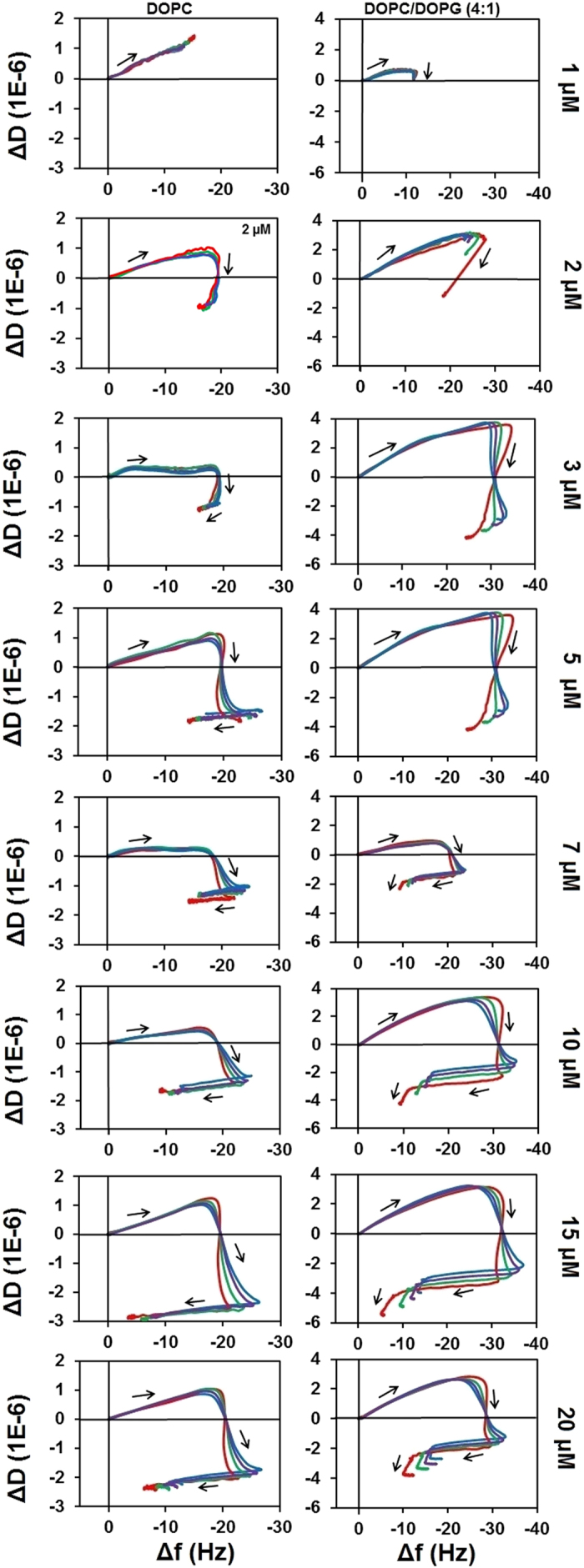
f-D plots of LL-37 with unsaturated membranes at varied peptide concentrations. The effect is shown for the third (red), fifth (green), seventh (purple), and ninth (blue) eigenmodes of the sensor chip oscillation.

The most apparent difference in the viscoelastic fingerprints in the cholesterol containing membranes is the replacement of the smooth “hook” by a more angular fingerprint with distinct stages. This suggests that the viscoelastic properties of the hydrophobic core have an effect on the LL-37 action; mixtures of the unsaturated phospholipids 1,2-dioleoyl-sn-glycerol-3-phosphocholine (DOPC) and 1,2-dioleoyl-sn-glycerol-3-phosphocholine/1,2-dioleoyl-sn-glycero-3-phospho-rac-(1-glycerol) (DOPC/DOPG) were explored to investigate these interactions. Figure 3 shows the effect of LL-37 on neat DOPC and a DOPC:DOPG (4:1) mixture. As a characteristic difference to their saturated equivalents, in both unsaturated lipid mixtures, LL-37 binding is a dissipative process already at 1 μM. At 3 μM second and third stages are observable, although in DOPC:DOPG (4:1) the third stage only becomes distinct at 7 μM. Importantly, at 7 μM the amount of binding (maximum frequency change) decreased below the Δf value observed for 5 μM. As opposed to the “hook” fingerprint of the saturated lipids (with the exception of DMPC: cholesterol (9:1)), distinct second and third stages can be observed. Hence, based on the QCM fingerprints, the key characteristics of LL-37 action do not depend on head-group charge, but rather on differences in the hydrophobic core, where the addition of cholesterol to saturated lipids resulted in nearly identical fingerprints to the unsaturated lipids.

Importantly, conducting the experiments above the DMPC phase transition temperature did not alter these characteristics, suggesting that the difference is not related to membrane fluidity. These results shed light on the controversy surrounding the head-group selectivity of LL-37; most previous studies did not consider the hydrophobic moieties as an important variable in the mechanism of action, and thus the physicochemical nature (i.e. length and unsaturation) of the aliphatic tail was not controlled^13^. On the other hand our results are consistent with prior observations questioning the role of headgroup charge in the LL-37 mechanism of action^18^.

## Membrane Modulating Action

**Figure 4:**
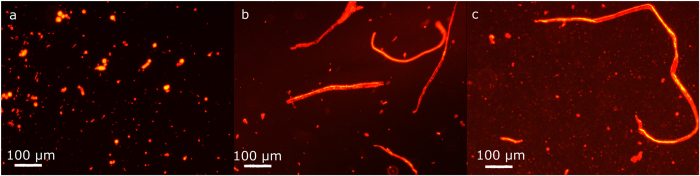
Fluorescence microscopy images of the effect of 10 μM LL-37 on saturated lipids. (**a**) DMPC liposomes before peptide injection; (**b**) after peptide injection. (**c**) DMPC:DMPG (4:1) lipid mixture after the addition of 10 μM LL-37.

The effects of LL-37 on different types of membranes were studied using wide-field epifluorescence microscopy. Liposomes labeled with a lipid-conjugated dye (red) and loaded with carboxyfluorescein (CF - green) solution are shown in Fig. 4 and Ext. Data Fig. 2; the co-localization of the two dyes appears yellow due to additive color mixing. Figure 4 shows the effect of 10 μM LL-37 on neat DMPC and DMPC:DMPG (4:1) liposomes. No intensity loss was observed over time suggesting that the membrane remains intact and the label is highly photostable (Ext. Data Fig. 2 - the UV lamp was shuttered between images). However, a slow aggregation process was observed that over 3 hours lead to a sudden transformation of the liposome clusters to tubular superstructures (Fig. 4(b)). DMPC:DMPG (4:1) formed similar tubular superstructures as neat DMPC (Fig. 4(c)). However, when unsaturated lipids were exposed to the same peptide concentration, no aggregation or tube formation was observed (data not shown).

Hence, the differences in QCM viscoelastic fingerprints lead to a visually different morphological effect: the peptides appear to facilitate liposome aggregation and fusion into tubular superstructures in case of saturated lipids, whereas no such process is observed in the case of unsaturated lipids. The clustering of liposomes suggests that the peptides form a fibre-like structure that fosters the coalescence of individual liposome structures. These fibres may be visible by transmission electron microscopy.

## Peptide-Lipid Nanofibres Observed with Transmission Electron Microscopy

**Figure 5:**
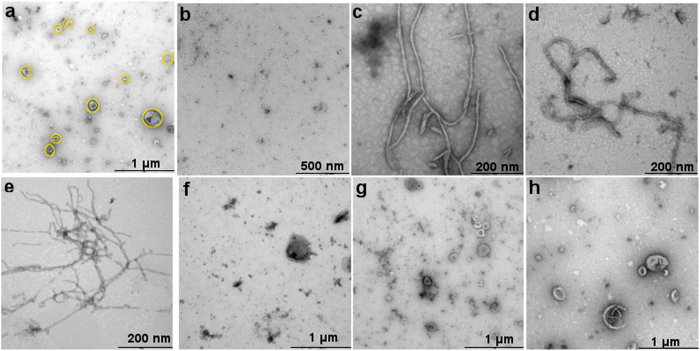
(**a**) TEM images of liposomes before peptide injection (yellow circles); (**b**) LL-37 alone; the effect of 10 μΜ LL-37 on (**c**) neat DMPC; (**d**) DMPC:DMPG (4:1); (**e**) DMPC:DMPG (3:2); (**f**) DMPC:cholesterol; (**g**) DOPC; and (**h**) DOPC:DOPG (4:1).

TEM imaging results of liposome-peptide interaction are depicted in Fig. 5. In the absence of peptides, the collapsed liposomes show the characteristic “wrinkled” morphology (Fig. 5(a)). Peptides in the absence of lipid (Fig. 5(b)) do not show fibrous structures; however, it is feasible to assume that the salt bridge formation predicted by the MD simulations leads to the aggregation visible in the image. Saturated lipids (DMPC, DMPC:DMPG (4:1) & (3:2)) exposed to LL-37 (Fig. 5(c,d)) exhibit fibre formation with a diameter in the order of 10 nm. Such fibres forming between liposomes in solution are the likely cause of the aggregation observed in fluorescence imaging (Ext. Data Fig. 2). In contrast, LL-37 does not cause observable structural change in unsaturated lipids (DOPC & DOPC:DOPG, Fig. 5(g,h)). Importantly, the morphological effect of LL-37 on DMPC:cholesterol is the same as that on unsaturated lipids Fig. 5(f). Hence TEM results illustrate that LL-37 has the same membrane modulating action mechanism on saturated fatty acids and a different, non-membrane-modulating interaction mechanism with unsaturated fatty acids and DMPC:cholesterol. These results are consistent with the conclusions of the QCM fingerprinting analysis and fluorescence microscopy.

It has been suggested before that LL-37 might exert its antimicrobial activity by a mechanism involving cytotoxic oligomers, similarly to amyloidogenic peptides in the presence of acidic phospholipids^31^. Our results suggest the formation of fibrous structures that form selectively with saturated lipids. Lipid-peptide composite fiber formation is seldom observed and is characteristic of larger AMPs, such as defensins^41^ where the fiber formation is the result of specific interaction with lipid headgroups. In most cases, polypeptide fibers are amyloid structures that require the refolding of the peptides from α-helices into β-strands. Hence, the folding of the peptides was also assessed.

## LL-37 Forms Fibres in α-Helical Conformation

The circular dichroism (CD) spectra of LL-37 both in buffer and in the presence of membranes is dominated by double minima at 208 and 222 nm (Ext. Data Fig. 3), characteristic of an α-helical secondary structure^42^. The presence of an isodichroic point (202–204 nm) indicates two state helix-coil equilibrium^32^. The α-helical content for LL-37 in PBS buffer was estimated to be 34%, in good agreement with previous reports^43^. The helical conformation of LL-37 is increased upon binding of the peptide to the membranes (Table 1), which is common for lipid-binding peptides^42^. In general, LL-37 adopts more helical conformation with saturated lipids.

The stability of the α-helical form of LL-37 on the membrane over time was also monitored (Ext. Data Fig. 4.). With DMPC the α-helicity of LL-37 did not change after 2 hours; the helicity was increased in the presence of DOPC, however, it also did not change after 2 hours. Accordingly, LL-37 is bound to the membrane in α-helical form, consistent with earlier literature reports^12^,^24^. Hence the formation of fibres does not involve the refolding of the peptide and the fibres are not amyloid-like since no significant β-structure is observed that would be characteristic of an amyloidogenic peptide^44^.

## LL-37 Causes Dye Leakage in Unsaturated Membranes

Thus far the membrane modulating effect of LL-37 with saturated lipids has been clearly demonstrated. However, QCM suggests that membrane disruption also takes place in case of unsaturated and cholesterol containing membranes. To confirm permeabilization of the membrane, dye leakage experiments have been carried out in a plate reader. As shown in Ext. Data Fig. 5(a) exposing neat DMPC liposomes to LL-37 lead to a slight decrease in the fluorescence intensity, likely due to light scattering on the peptide molecules; the same was observed for DMPC/DMPG (4:1) (data not shown). The photobleaching of the CF dye was negligible (Ext. Data Fig. 5(c)). In contrast, after introducing LL-37 to dye loaded DOPC liposomes, there is an immediate ~4 fold increase of the fluorescence intensity, indicating membrane permeabilization. Hence, the effect of LL-37 on DOPC liposomes is consistent with a pore forming mechanism as proposed in previous studies^17^,^24^.

In those cases where instantaneous dye release had not been observed, the samples were monitored for extended periods of time. Correlating the dye leakage trend to fluorescence microscopy observations for DMPC membranes (Ext. Data Fig. 6) it is apparent that after 2 hours there is a sudden dye release event, suggesting membrane disintegration that is followed by the formation of the large tubular structures observed in optical microscopy (Ext. Data Fig. 6(c,d)). Such behaviour has never been observed for an antimicrobial peptide before.

Previous studies did not arrive at a decisive conclusion about the lipid specificity of LL-37. However, in these earlier studies only the role of lipid head-groups was studied, whereas our results here suggest that specificity, and hence the mode of action, is defined by the structure and composition of the hydrophobic membrane core, without any perceivable contribution from the head-groups. The biophysical results are in good agreement with the known biological roles of LL-37. Cathelicidin proteins are often expressed by cells in direct contact with the environment^30^. LL-37 is present in low concentration at the sites of potential bacterial intrusion (i.e. mucous membranes) as an innate immunity peptide, and is also released in large amounts at sites of injury where it has the dual role of eliminating bacteria and contributing to wound healing. We propose that the fibre formation is a secondary defence mechanism that recruits lipid molecules to form a protective “armour” against bacterial attack. Thus, in terms of antimicrobial specificity, LL-37 exhibits a hitherto unknown mechanism based on a dual-offensive protective role.

## Conclusions

LL-37 exhibited two distinct modes of action, as a pore former in unsaturated or cholesterol containing lipids, causing immediate dye release, and as a membrane modulating agent in saturated lipids, causing the formation of tubular and fibrillar peptide-lipid superstructures. The head-group charge did not affect the mode of action, although phosphatidyl glycerol residues might facilitate the separation of peptide aggregates upon membrane binding. While some aggregation was evident from intensity loss in CD measurements, our results suggest that the mixed aggregation (fibre formation) of LL-37 with lipidic species is a preferential aggregation pathway. These results explain the contradictory data in prior reports while also opening new avenues for the design of peptide antimicrobial agents.

## Materials and Methods

Potassium dihydrogen phosphate (KH_2_PO_4,_ ACS reagent), potassium hydrogen phosphate (K_2_HPO_4,_ ACS reagent), 3-mercaptopropionic acid (MPA; HPLC grade >99%) were purchased from Fluka Bio-Chimia (Switzerland). Sodium chloride (NaCl) was purchased from Merck (Darmstadt, Germany). Propan-2-ol, hydrogen peroxide (H_2_O_2_), chloroform (ACS Reagent, 99.8%), ammonium hydroxide solution (28%, Analytical Univar Reagent), methanol (>99.9%, spectrophotometric grade) and ethanol (HPLC/spectrophotometric grade) were purchased from Sigma-Aldrich (Castel hill, NSW, Australia). LL-37 was purchased from GLBiochem (China) and the molecular weight was confirmed with mass spectrometry. 1,2-dimyristoyl-sn-glycero-3-phosphocholine (DMPC), the sodium salt of 1,2-dimyristoyl-sn-glycero-3-phosphoglycerol (DMPG), 1,2-dioleoyl-sn-glycerol-3-phosphocholine (DOPC), the sodium salt of 1,2-dioleoyl-sn-glycero-3-phospho-rac-(1-glycerol) (DOPG), and cholesterol were purchased from Avanti Polar Lipids (Alabaster, AL, USA). 5(6)-carboxyfluorescein (CF) was purchased from Sigma-Aldrich (USA) and DMPE-atto-594 was purchased from ATTO-TEC GmbH (Germany).

Lipids were dissolved in chloroform; for DMPG and DOPG, 3% methanol was added to improve solubility. Desired ratios of lipids: neat DMPC, DMPC/DMPG (4:1), (3:2), DMPC/cholesterol (9:1), neat DOPC, and DOPC/DOPG (4:1), were measured into round bottom glass test tubes. The solvent was evaporated under a gentle stream of N_2_ and dried overnight. Liposomes were formed by hydration in 20 mM phosphate buffered saline (100 mM NaCl at pH 6.9).

### Quartz Crystal Microbalance

Quartz Crystal Microbalance “with Dissipation Monitoring” (QCM) measurements were performed with a Q-SENSE E4 system (Q-Sense, Sweden). The sensor crystals used were 5 MHz, AT-cut, polished quartz disks with evaporated gold sensor surface. The changes of resonance frequency (Δf) and energy dissipation (ΔD) upon mass deposition were measured simultaneously at four different eigenmodes (3rd, 5th, 7th and 9th) of the crystal resonance. Sensor chips were rinsed with ethanol and dried under a gentle stream of N_2_ gas, and then placed into a 3:1:1 mixture of 18.2 MΩ cm deionized water (Ultrapure, Sartorius AG, Germany), hydrogen peroxide (30% solution) and ammonium hydroxide (28-30% solution) for 20 min at 70 °C; the chips were then rinsed with deionized water and dried. The clean gold surface was treated with 2% (w/w) MPA in propan-2-ol overnight to form a self-assembled monolayer as a support for the biomimetic membrane^45^. The next day chips were rinsed in propan-2-ol for 5 min to remove excess MPA and then assembled into the QCM chambers. At first water was injected into the chambers to hydrate the surface of the chips and then assay buffer was introduced. QCM experiments were repeated at least 3 times for each peptide and at each concentration (i.e. N = 3). All experiments were performed following an established protocol that involved flushing 1 ml of liposome solution through the measurement chamber at 100 μL/min and at 19 °C^35^. Peptides were injected into the QCM chamber following the formation of the single bilayer: 13–15 Hz frequency and 2.5–3.0 × 10^−6^ dissipation change was confirmed^28^,^46^. Control experiments on MPA coated gold confirmed the absence of peptide binding. Hence, all measured structural changes correspond to the interaction between peptide and the membranes.

### QCM Fingerprinting

Plotting the dissipation change against frequency change (f-D curve) for a biomolecular interaction process can be used to provide a mechanistic fingerprint, an approach first introduced by Rodahl *et al*.^47^ and successfully used for the study of AMP-membrane interactions^29^,^35^. A detailed description was provided in a previous work^29^. Briefly, the f-D curve correlates viscoelastic changes to mass uptake, which reflect changes in the membrane organization^35^,^48^. Thus, each direction in f-D space reveals distinct mechanical changes. The direction [−f, +D] shows mass uptake; conversely an interaction “vector” pointing towards [+f, −D] reflects a process in which mass is lost^37^, while vectors normal to these directions, [−f, −D] and [+f, +D], reveal structural changes in the deposited material^29^.

### Fluorescence Microscopy Imaging

Liposomes were loaded with 10 mM aqueous solution of 5(6)-carboxyfluorescein (CF) (λ_fl_ = 517 nm) by rehydrating the dry lipids in the aqueous solution of the fluorophore. Co-labelling was also employed by mixing 0.1% DMPE-atto-594 (λ_fl_ = 627 nm) with the lipids in an organic solvent following the protocol described above, and the liposomes formed from these membranes were loaded with CF. The co-localization of the two dyes in intact liposomes yields a yellow colour. Excess 5(6)-carboxyfluorescein was removed *via* dialysis. The co-labelled liposome suspension was stored in dark and it was stable over a period of 2–4 days. Experiments were performed with a Nikon Eclipse TM100 epifluorescence microscope equipped with a DS-Fi1 colour CCD camera, using a UV lamp excitation source. Images were taken through a QCM window cell during *in situ* membrane deposition. Importantly, this setup requires long working distance objectives and is not compatible with confocal microscopy. The setup gives a slight aberration due to the tilt of the glass cover of the QCM cell in relation to the optical axis of the microscope objective.

### Transmission Electron Microscopy (TEM)

Neat LL-37 as well as membrane mixtures with 10 μM LL-37 (10 μL) were applied for 2 min to 400-mesh copper grids (ProSciTech) coated with a thin layer of carbon. Excess material was removed by blotting, and samples were negatively stained twice with 10 μL of a 2% (wt/vol) uranyl acetate solution (Electron Microscopy Sciences, Hatfield PA, USA). The grids were air-dried and viewed using a JEOL JEM-2010 transmission electron microscope operated at 80 kV.

### Circular Dichroism Spectroscopy

CD spectra from 200 to 250 nm were recorded with a Model 420 CD spectrophotometer (AVIV, USA) with the temperature maintained at 37 °C. A 1-mm path length quartz cell was used at a final concentration of 10 μM peptide and 100 μM lipid in PBS buffer. Each experiment was repeated three times. Interference from the circular differential scattering by liposomes was eliminated by subtracting CD spectra of liposome suspension recorded in the absence of peptide. Data are shown as mean residue molar ellipticity (deg cm^−1^ dmol^−1^). The percentage of α-helical content was estimated from the molar ellipticity at 222 nm (θ_222_) using equation 1^49^.


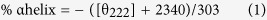


### Dye Leakage

Dye release upon peptide interaction was monitored using 5(6)-carboxyfluorescein (CF) loaded vesicles. Liposomes were prepared as described above while lipids were hydrated in a buffer containing 10 mM CF. Dye was removed from the medium by dialysis. Leakage of CF from liposomes was detected as an increase in fluorescence intensity, monitored using Spectramax M5 spectrophotometer (Molecular Devices, Silicon Valley, CA, USA) with excitation wavelength of 480 nm while the emission was monitored in a range of 500–530 nm.

### Simulations

Molecular dynamics (MD) simulations were carried out using the GROMACS package (vers. 5.1)^50^ and GROMOS force field (vers. 53a6)^51^. Ionizable residues were assumed to be in their standard state at neutral pH. Twenty-four copies of the LL-37 peptide were placed in a 180 Å cubic box to mimic the effect of concentration. The box was filled with SPC^52^ water molecules and the system was kept neutral by the addition of chlorine counterions. Proteins and solvent were coupled separately to a thermal bath at 300 K using a velocity-rescale thermostat^53^; a Berendsen barostat^54^ at 1 bar with a coupling time of 0.5 ps was also applied. All simulations were performed with a single nonbonded cutoff of 10 Å, applying a neighbour list update frequency of 10 steps (20 fs). The particle mesh Ewald method^55^ was used to account for long-range electrostatics, applying a grid width of 1.2 Å and a fourth-order spline interpolation. Bond lengths were constrained using the LINCS algorithm^56^.

All simulations consisted of an initial minimization of water molecules followed by 200 ps of MD simulations with the protein fixed. After removing all the restraints on the protein, MD simulations were continued for 50 ns. MD simulations were analysed using standard GROMACS tools. Figures were produced using VMD^55^.

## Additional Information

**How to cite this article**: Shahmiri, M. *et al*. Membrane Core-Specific Antimicrobial Action of Cathelicidin LL-37 Peptide Switches Between Pore and Nanofibre Formation. *Sci. Rep.* **6**, 38184; doi: 10.1038/srep38184 (2016).

**Publisher's note:** Springer Nature remains neutral with regard to jurisdictional claims in published maps and institutional affiliations.

## Supplementary Material

Supplementary Information

## Figures and Tables

**Table 1:** The percentage α-helicity of LL-37 in PBS and various membrane environments.

Membrane	Helicity%
- - - -	34.47
DMPC	60.01
DMPC/DMPG (4:1)	71.57
DMPC/Cholesterol	53.83
DMPC/DMPG (3:2)	79.60
DOPC	52.34
DOPC/DOPG (4:1)	67.67

## References

[b1] Tonks A. Drug resistance is a worldwide threat, warns report. BMJ 309, 1109, doi: 10.1136/bmj.309.6962.1109 (1994).7987100

[b2] Boucher H. W. . Bad bugs, no drugs: no ESKAPE! An update from the Infectious Diseases Society of America. Clin. Infect. Dis. 48, 1–12, doi: 10.1086/595011 (2009). 19035777

[b3] Hancock R. E. & Sahl H.-G. Antimicrobial and host-defense peptides as new anti-infective therapeutic strategies. Nat. Biotechnol. 24, 1551–1557, doi: 10.1038/nbt1267 (2006). 17160061

[b4] Zasloff M. Antimicrobial peptides of multicellular organisms. Nature 415, 389–395, doi: 10.1038/415389a (2002). 11807545

[b5] Nizet V. . Innate antimicrobial peptide protects the skin from invasive bacterial infection. Nature 414, 454–457, doi: 10.1038/35106587 (2001). 11719807

[b6] Zanetti M. Cathelicidins, multifunctional peptides of the innate immunity. J. Leukoc. Biol. 75, 39–48, doi: 10.1189/jlb.0403147 (2004). 12960280

[b7] Sørensen O. E. . Human cathelicidin, hCAP-18, is processed to the antimicrobial peptide LL-37 by extracellular cleavage with proteinase 3. Blood 97, 3951–3959, doi: 10.1182 (2001). 11389039 10.1182/blood.v97.12.3951

[b8] Joly S., Maze C., McCray P. B. Jr. & Guthmiller J. M. Human β-Defensins 2 and 3 Demonstrate Strain-Selective Activity against Oral Microorganisms. J. Clin. Microbiol. 42, 1024–1029, doi: 10.1128/JCM.42.3.1024-1029.2004 (2004). 15004048 PMC356847

[b9] Turner J., Cho Y., Dinh N. N., Waring A. J. & Lehrer R. I. Activities of LL-37, a cathelin-associated antimicrobial peptide of human neutrophils. Antimicrob. Agents Chemother. 42, 2206–2214 (1998). 9736536 10.1128/aac.42.9.2206 PMC105778

[b10] Henzler Wildman K. A., Lee D.-K. & Ramamoorthy A. Mechanism of lipid bilayer disruption by the human antimicrobial peptide, LL-37. Biochemistry 42, 6545–6558, doi: 10.1021/bi0273563 (2003). 12767238

[b11] Li X., Li Y., Han H., Miller D. W. & Wang G. Solution structures of human LL-37 fragments and NMR-based identification of a minimal membrane-targeting antimicrobial and anticancer region. J. Am. Chem. Soc. 128, 5776–5785, doi: 10.1021/ja0584875 (2006). 16637646

[b12] Porcelli F. . NMR structure of the cathelicidin-derived human antimicrobial peptide LL-37 in dodecylphosphocholine micelles. Biochemistry 47, 5565–5572, doi: 10.1021/bi702036s (2008). 18439024 PMC5873590

[b13] Neville F. . Lipid headgroup discrimination by antimicrobial peptide LL-37: Insight into mechanism of action. Biophys. J. 90, 1275–1287, doi: 10.1529/biophysj.105.067595 (2006). 16299073 PMC1367279

[b14] Shai Y. Mechanism of the binding, insertion and destabilization of phospholipid bilayer membranes by α-helical antimicrobial and cell non-selective membrane-lytic peptides. Biochim. Biophys. Acta 1462, 55–70, doi: 10.1016/S0005-2736(99)00200-X (1999). 10590302

[b15] Yang L., Harroun T. A., Weiss T. M., Ding L. & Huang H. W. Barrel-stave model or toroidal model? A case study on melittin pores. Biophys. J. 81, 1475–1485, doi: 10.1016/S0006-3495(01)75802-X (2001). 11509361 PMC1301626

[b16] Brogden K. A. Antimicrobial peptides: pore formers or metabolic inhibitors in bacteria? Nat. Rev. Microbiol. 3, 238–250, doi: 10.1038/nrmicro1098 (2005). 15703760

[b17] Wildman K. A. H., Lee D. K. & Ramamoorthy A. Mechanism of lipid bilayer disruption by the human antimicrobial peptide, LL-37. Biochemistry 42, 6545–6558, doi: 10.1021/bi0273563 (2003). 12767238

[b18] Sevcsik E. . Interaction of LL-37 with model membrane systems of different complexity: influence of the lipid matrix. Biophys. J. 94, 4688–4699, doi: 10.1529/biophysj.107.123620 (2008). 18326643 PMC2397346

[b19] Larrick J. W., Hirata M., Zhong J. & Wright S. C. Anti-microbial activity of human CAP18 peptides. Immunotechnology 1, 65–72, doi: 10.1016/1380-2933(95)00006-2 (1995). 9373334

[b20] Tanaka D., Miyasaki K. & Lehrer R. Sensitivity of Actinobacillus actinomycetemcomitans and Capnocytophaga spp. to the bactericidal action of LL‐37: a cathelicidin found in human leukocytes and epithelium. Oral Microbiol. Immunol. 15, 226–231, doi: 10.1034/j.1399-302x.2000.150403.x (2000). 11154407

[b21] Henzler-Wildman K. A., Martinez G. V., Brown M. F. & Ramamoorthy A. Perturbation of the hydrophobic core of lipid bilayers by the human antimicrobial peptide LL-37. Biochemistry 43, 8459–8469, doi: 10.1021/bi036284s (2004). 15222757

[b22] Oren Z., Lerman J. C., Gudmundsson G. H., Agerberth B. & Shai Y. Structure and organization of the human antimicrobial peptide LL-37 in phospholipid membranes: Relevance to the molecular basis for its non-cell-selective activity. Biochem. J. 341, 501–513, doi: 10.1042/0264-6021:3410501 (1999). 10417311 PMC1220385

[b23] Barns K. J. & Weisshaar J. C. Real-time attack of LL-37 on single Bacillus subtilis cells. Biochim. Biophys. Acta 1828, 1511–1520, doi: 10.1016/j.bbamem.2013.02.011 (2013). 23454084 PMC3625923

[b24] Lee C.-C., Sun Y., Qian S. & Huang H. W. Transmembrane Pores Formed by Human Antimicrobial Peptide LL-37. Biophys. J. 100, 1688–1696, doi: 10.1016/j.bpj.2011.02.018 (2011). 21463582 PMC3072607

[b25] Byfield F. J. . Cathelicidin LL-37 increases lung epithelial cell stiffness, decreases transepithelial permeability, and prevents epithelial invasion by Pseudomonas aeruginosa. J. Immunol. 187, 6402–6409 (2011). 22095714 10.4049/jimmunol.1102185

[b26] Larrick J. W. . Antimicrobial activity of rabbit CAP18-derived peptides. Antimicrob. Agents Chemother. 37, 2534–2539, doi: 10.1128/AAC.37.12.2534 (1993). 8109914 PMC192730

[b27] Isogai E. . Sensitivity of genera Porphyromonas and Prevotella to the bactericidal action of C‐terminal domain of human CAP18 and its analogues. Oral Microbiol. Immunol. 18, 329–332, doi: 10.1034/j.1399-302X.2003.00083.x (2003). 12930528

[b28] Hasan I. Y. & Mechler A. Viscoelastic changes measured in partially suspended single bilayer membranes. Soft Matter 11, 5571–5579, doi: 10.1039/C5SM00278H (2015). 26073288

[b29] Shahmiri M., Enciso M. & Mechler A. Controls and constrains of the membrane disrupting action of Aurein 1.2. Sci. Rep. 5, 16378, doi: 10.1038/srep16378 (2015). 26574052 PMC4648102

[b30] Vandamme D., Landuyt B., Luyten W. & Schoofs L. A comprehensive summary of LL-37, the factotum human cathelicidin peptide. Cell. Immunol. 280, 22–35, doi: 10.1016/j.cellimm.2012.11.009 (2012). 23246832

[b31] Sood R., Domanov Y., Pietiainen M., Kontinen V. P. & Kinnunen P. K. J. Binding of LL-37 to model biomembranes: Insight into target vs host cell recognition. Biochim. Biophys. Acta 1778, 983–996, doi: 10.1016/j.bbamem.2007.11.016 (2008). 18166145

[b32] Baldwin R. L. How Hofmeister ion interactions affect protein stability. Biophys. J. 71, 2056–2063, doi: 10.1016/S0006-3495(96)79404-3 (1996). 8889180 PMC1233672

[b33] Wang G., Mishra B., Epand R. F. & Epand R. M. High-quality 3D structures shine light on antibacterial, anti-biofilm and antiviral activities of human cathelicidin LL-37 and its fragments. Biochim. Biophys. Acta 1838, 2160–2172, doi: 10.1016/j.bbamem.2014.01.016 (2014). 24463069 PMC4082733

[b34] Fernandez D. I., Lee T.-H., Sani M.-A., Aguilar M.-I. & Separovic F. Proline facilitates membrane insertion of the antimicrobial peptide maculatin 1.1 via surface indentation and subsequent lipid disordering. Biophys. J. 104, 1495–1507, doi: 10.1016/j.bpj.2013.01.059 (2013). 23561526 PMC3617439

[b35] Praporski S., Mechler A., Separovic F. & Martin L. L. Subtle Differences in Initial Membrane Interactions Underpin the Selectivity of Small Antimicrobial Peptides. ChemPlusChem 80, 91–96, doi: 10.1002/cplu.201402318 (2015).

[b36] Mozsolits H., Thomas W. G. & Aguilar M. I. Surface plasmon resonance spectroscopy in the study of membrane‐mediated cell signalling. J. Pept. Sci. 9, 77–89, doi: 10.1002/psc.439 (2003). 12630693

[b37] McCubbin G. A. . QCM-D fingerprinting of membrane-active peptides. Eur. Biophys. J. 40, 437–446 (2011). 21161523 10.1007/s00249-010-0652-5

[b38] McMullen T. P. & McElhaney R. N. Physical studies of cholesterol-phospholipid interactions. Curr. Opin. Colloid Interface Sci. 1, 83–90, doi: 10.1016/S1359-0294(96)80048-3 (1996).

[b39] Shai Y. Mode of action of membrane active antimicrobial peptides. Biopolymers 66, 236–248, doi: 10.1002/bip.10260 (2002). 12491537

[b40] Wadhwani P. . Membrane-active peptides and the clustering of anionic lipids. Biophys. J. 103, 265–274, doi: 10.1016/j.bpj.2012.06.004 (2012). 22853904 PMC3400765

[b41] Poon I. K. . Phosphoinositide-mediated oligomerization of a defensin induces cell lysis. Elife 3, e01808, doi: 10.7554/eLife.01808 (2014). 24692446 PMC3968744

[b42] MacPhee C. E., Perugini M. A., Sawyer W. H. & Howlett G. J. Trifluoroethanol induces the self-association of specific amphipathic peptides. *FEBS Lett*. 416, 265–268, doi: 10.1016/S0014-5793(97)01224-6 (1997). 9373166

[b43] Xhindoli D. . New aspects of the structure and mode of action of the human cathelicidin LL-37 revealed by the intrinsic probe p-cyanophenylalanine. Biochem. J. 465, 443–457, doi: 10.1042/BJ20141016 (2015). 25378136

[b44] Griffin M. D. . Phospholipid interaction induces molecular-level polymorphism in apolipoprotein C-II amyloid fibrils via alternative assembly pathways. J. Mol. Biol. 375, 240–256, doi: 10.1016/j.jmb.2007.10.038 (2008). 18005990

[b45] Mechler A., Nawaratna G., Aguilar M.-I. & Martin L. L. A study of protein electrochemistry on a supported membrane electrode. Int. J. Pept. Res. Ther. 12, 217–224, doi: 10.1007/s10989-006-9029-0 (2006).

[b46] Mechler A. . Structure and homogeneity of pseudo-physiological phospholipid bilayers and their deposition characteristics on carboxylic acid terminated self-assembled monolayers. Biomaterials 30, 682–689, doi: 10.1016/j.biomaterials.2008.10.016 (2009). 19000635

[b47] Rodahl M. . Simultaneous frequency and dissipation factor QCM measurements of biomolecular adsorption and cell adhesion. Faraday Discuss. 107, 229–246, doi: 10.1039/a703137h (1997). 9569776

[b48] Hall K., Lee T.-H., Mechler A. I., Swann M. J. & Aguilar M.-I. Real-time measurement of membrane conformational states induced by antimicrobial peptides: balance between recovery and lysis. Sci. Rep. 4, doi: 10.1038/srep05479 (2014). PMC4073255 24969959

[b49] Perugini M. A., Schuck P. & Howlett G. J. Self-association of human apolipoprotein E3 and E4 in the presence and absence of phospholipid. J. Biol. Chem. 275, 36758–36765, doi: 10.1074/jbc.M005565200 (2000). 10970893

[b50] Abraham M. J. . GROMACS: High performance molecular simulations through multi-level parallelism from laptops to supercomputers. SoftwareX 1, 19–25, doi: 10.5281/zenodo.45175 (2015).

[b51] Oostenbrink C., Villa A., Mark A. E. & Van Gunsteren W. F. A biomolecular force field based on the free enthalpy of hydration and solvation: The GROMOS force‐field parameter sets 53A5 and 53A6. J. Comput. Chem. 25, 1656–1676, doi: 10.1002/jcc.20090 (2004). 15264259

[b52] Berendsen H., Grigera J. & Straatsma T. The missing term in effective pair potentials. J. Phys. Chem. 91, 6269–6271, doi: 10.1021/j100308a038 (1987).

[b53] Bussi G., Donadio D. & Parrinello M. Canonical sampling through velocity rescaling. J. Chem. Phys. 126, 014101, doi: 10.1063/1.2408420 (2007). 17212484

[b54] Berendsen H. J., Postma J. P. M., van Gunsteren W. F., DiNola A. & Haak J. Molecular dynamics with coupling to an external bath. J. Chem. Phys. 81, 3684–3690, doi: 10.1063/1.448118 (1984).

[b55] Darden T., Perera L., Li L. & Pedersen L. New tricks for modelers from the crystallography toolkit: the particle mesh Ewald algorithm and its use in nucleic acid simulations. Structure with Folding and Design 7, R55–R60, doi: 10.1016/S0969-2126(99)80033-1 (1999). 10368306

[b56] Hess B. P-LINCS: A parallel linear constraint solver for molecular simulation. J. Chem. Theory Comput. 4, 116–122, doi: 10.1021/ct700200b (2008). 26619985

