# Supplementary material for: Membrane Core-Specific Antimicrobial Action of Cathelicidin LL-37 Peptide Switches Between Pore and Nanofibre Formation
Source: Sci Rep. 2016 Nov 30;6:38184. doi: 10.1038/srep38184 (PMC5128859; doi:10.1038/srep38184)
Supplement: Supplementary Information [file srep38184-s1.pdf]

Extended Data for

Membrane Core-Specific Antimicrobial Action of Cathelicidin LL-37 Peptide Switches Between Pore and Nanofibre Formation

Mahdi Shahmiri, Marta Enciso, Christopher G. Adda, Brian J. Smith, Matthew A. Perugini and Adam Mechler

La Trobe Institute for Molecular Science, La Trobe University, Australia

All sensograms for the f-D fingerprints shown in the article:

A

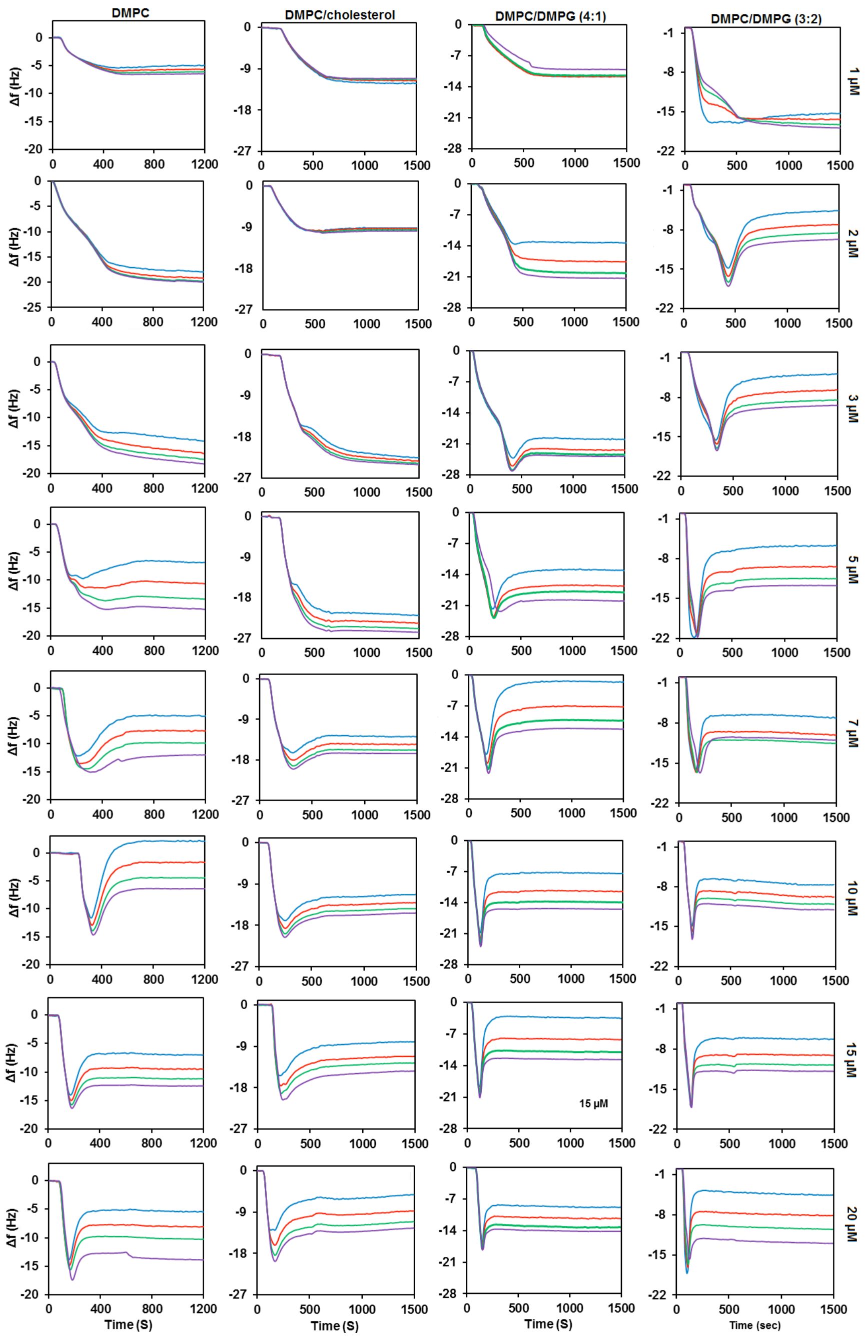

B

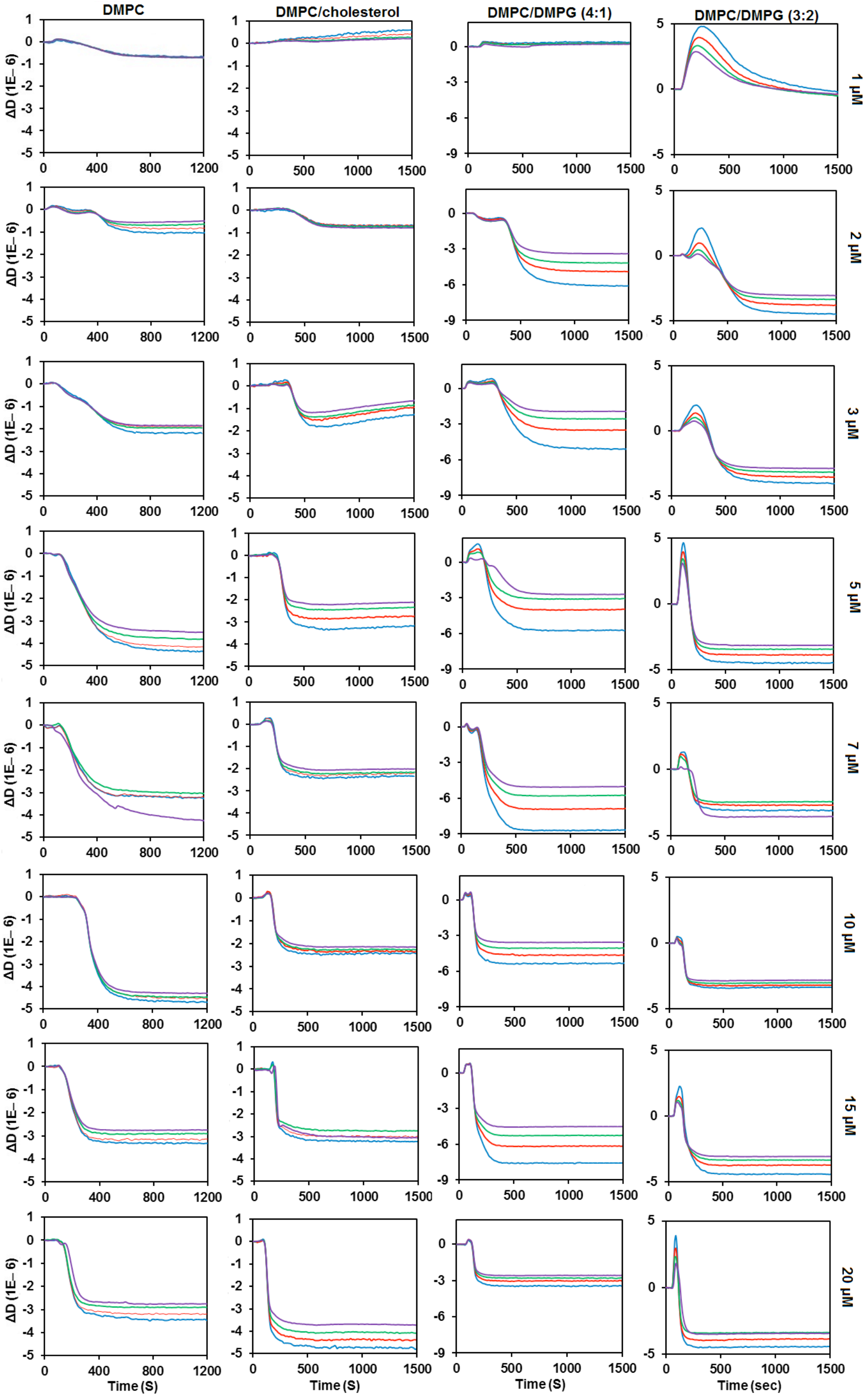
DMPC
DMPC/cholesterol
DMPC/DMPG (4:1)
DMPC/DMPG (3:2)
ΔD (1E– 6)
1 μM
2 μM
3 μM
5 μM
7 μM
10 μM
15 μM
20 μM
Time (S)
Time (sec)

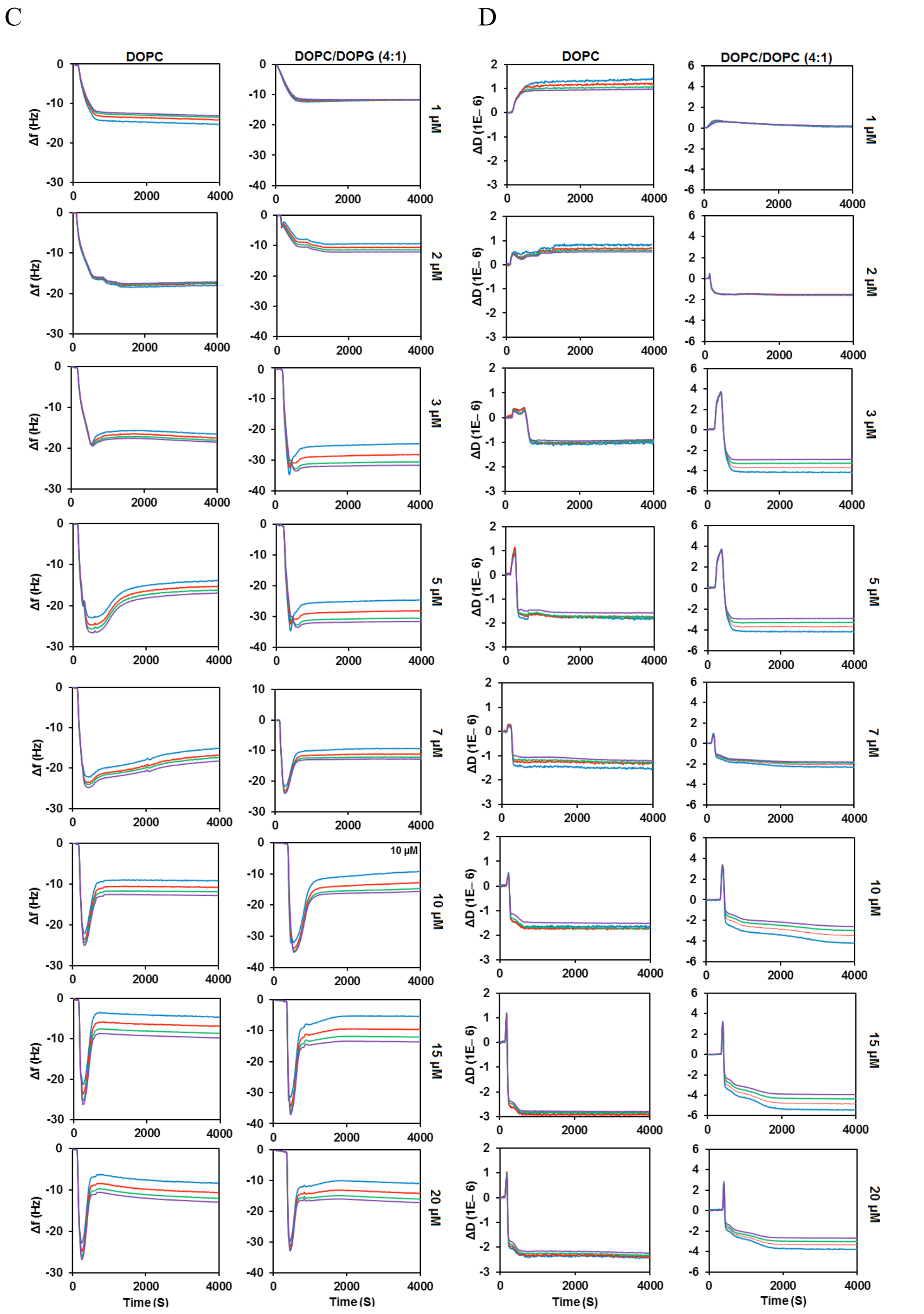

Extended Data Figure 1. QCM sensograms of frequency and dissipation signals plotted against time for all f-D fingerprints included in the article. (A) f-t sensograms of saturated lipids; (B) D-t sensograms of saturated lipids; (C) f-t sensograms of unsaturated lipids; (D) D-t sensograms of unsaturated lipids.

Fluorescence microscopy imaging

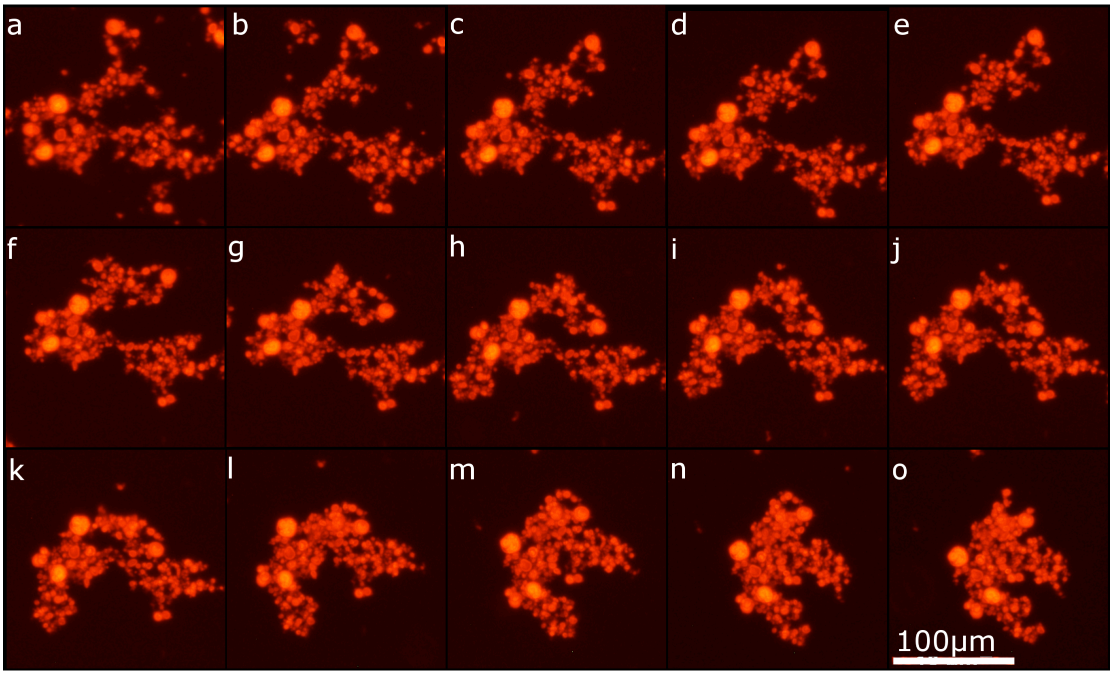

Extended Data Figure 2. Fluorescence microscopy imaging shows the aggregation of DMPC liposomes upon addition of LL-37.

CD measurements

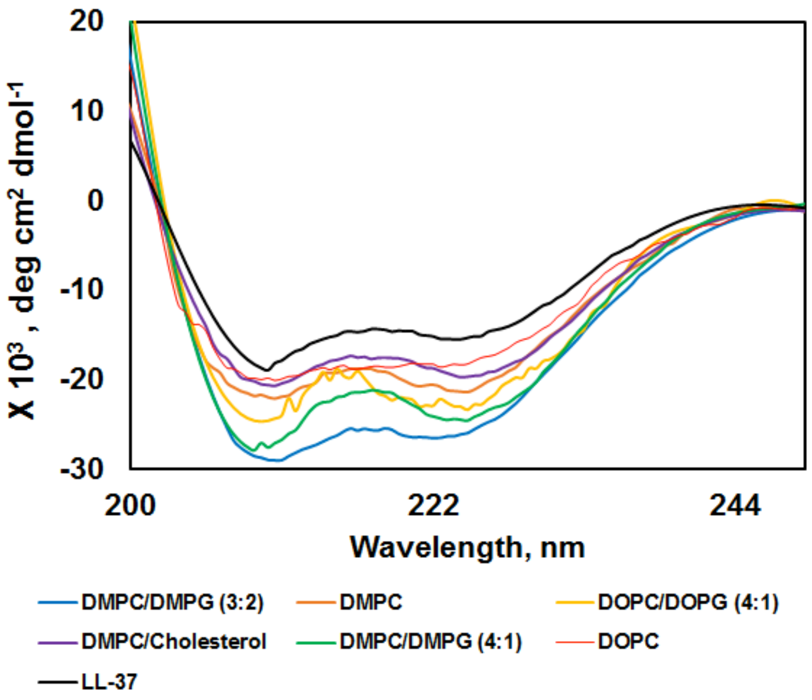

Extended Data Figure 3. Circular dichroic spectra of 10 μM LL-37 in buffer and in the presence of different membrane compositions as indicated.

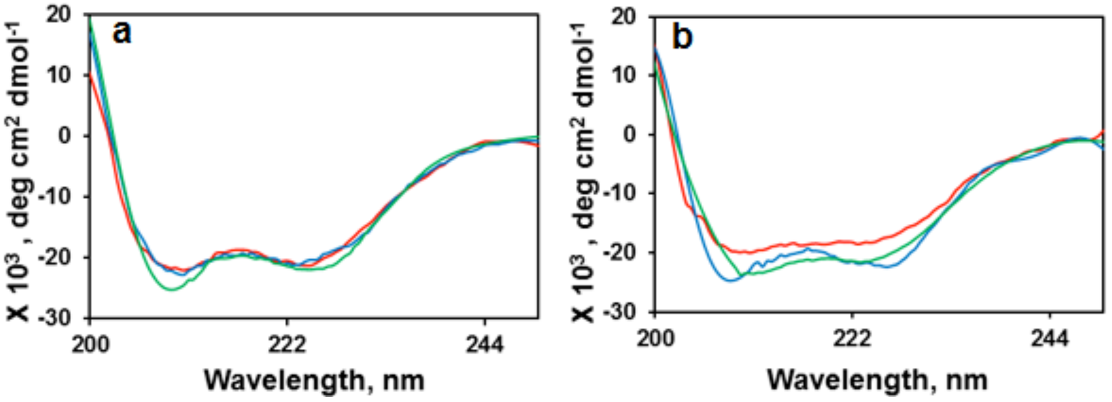

Extended Data Figure 4. Circular dichroic spectra of (a) neat DMPC and (b) DOPC over time. After peptide injection (red), after 1 h (green), and after 2 h (blue).

Dye leakage experiments

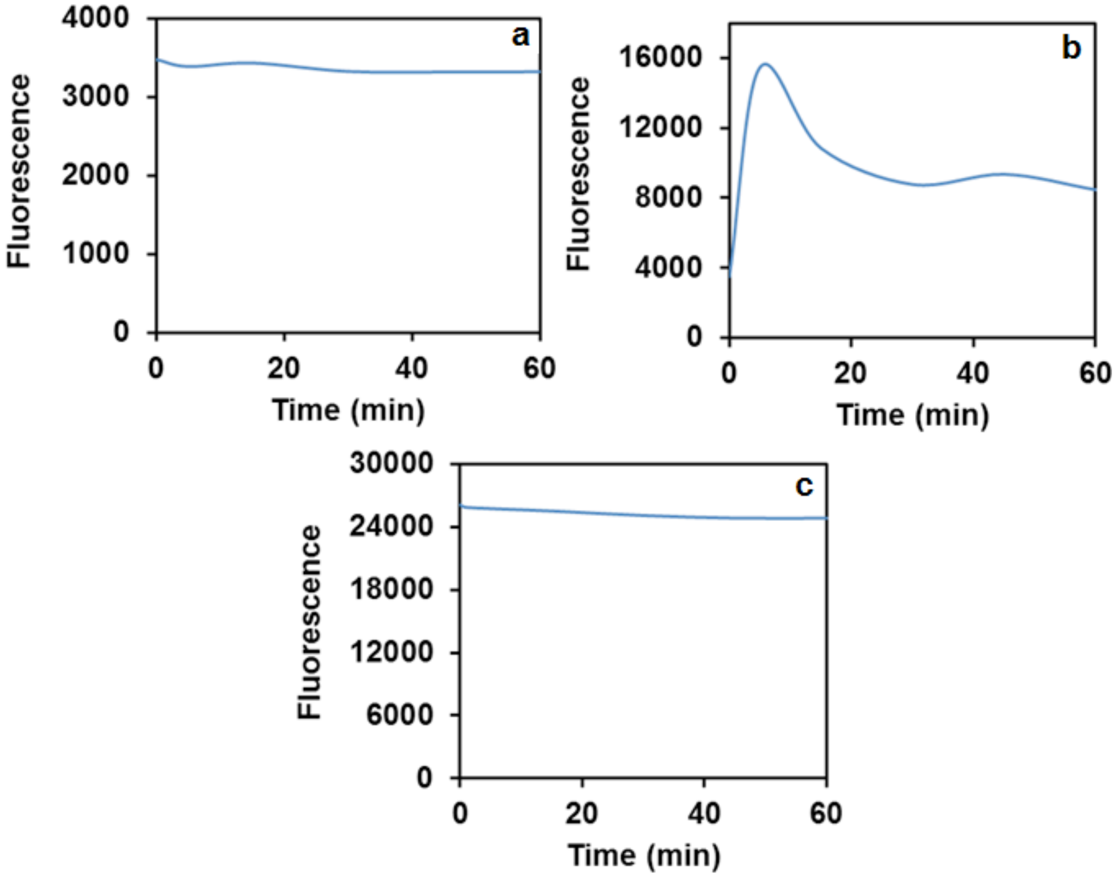

Extended Data Figure 5. Dye leakage experiments of (a) DMPC, (b) DOPC liposomes with 10 µM LL-37. (c) control: 5(6)-carboxyfluorescein solution

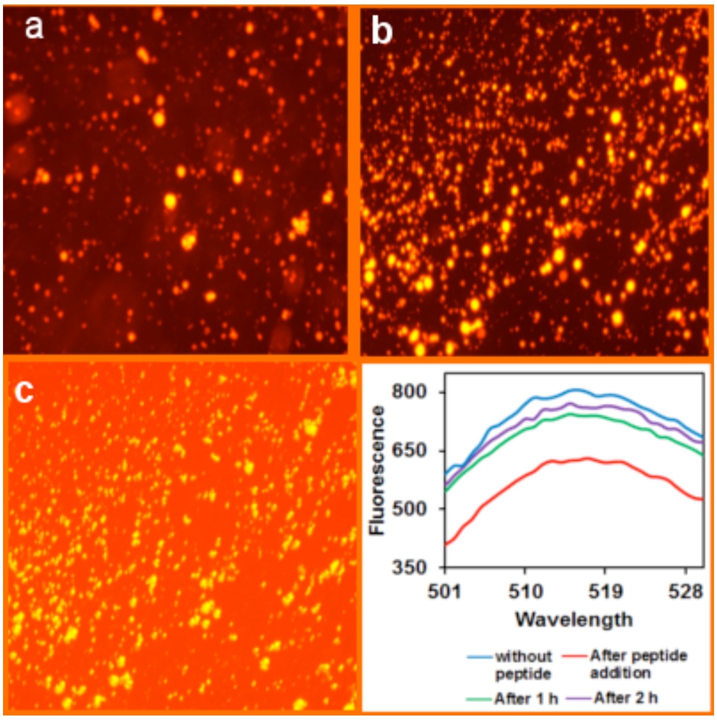

Extended Data Figure 6. (a) Fluoresence images of (a) afetr peptide addition, (b) after 1 h, and (c) after 2h; (d), fluorescent intensity (dye leakage) spectra recorded over 2 hours.
